# Test your knowledge and understanding

**Published:** 2018-11-09

**This quiz is designed to help you test your own understanding of the concepts covered in this issue, and to reflect on what you have learnt.**

**Figure:**
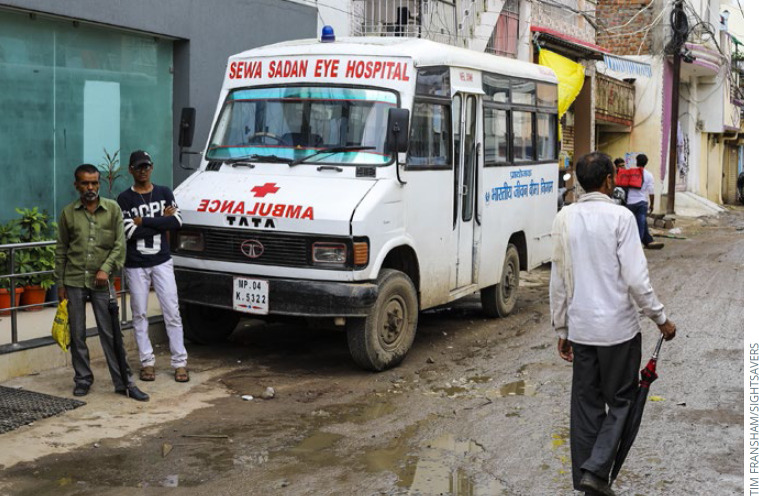
It is important to see patients quickly in eye emergencies. INDIA

We hope that you will also discuss the questions with your colleagues and other members of the eye care team, perhaps in a journal club. To complete the activities online – and get instant feedback – please visit **www.cehjournal.org**

**Question 1 In ophthalmic emergencies, which of the following statements are true?** *Tick all that are true.*
  □ **a.** Dealing with emergencies is the ophthalmologist's responsibility and other members of staff should just follow instructions
  □ **b.** It is not helpful to practise for emergencies, because simulation is not like the real thing
  □ **c.** Eye emergencies must be referred to a specialist immediately
  □ **d.** Although uncommon, everyone encounters an ophthalmic emergency at some time
**Question 2 A 60-year-old farmer attends your clinic complaining of rapid loss of vision and a painful red eye. On examination his vision is CF and the eye is red, but the lids are not swollen. Which of the following are possible diagnoses?** *Tick all that are true.*
  □ **a.** Retinal detachment
  □ **b.** Acute glaucoma
  □ **c.** Microbial keratitis
  □ **d.** Orbital cellulitis
  □ **e.** Optic nerve compression
**Question 3 Managing emergency infections: which of the following are true?** *Tick all that are true.*
  □ **a.** Treatment of endophthalmitis should be delayed until you have identified the infectious organism
  □ **b.** Orbital cellulitis can be treated with intensive topical antibiotics
  □ **c.** The most severe form of ophthalmia neonatorum is caused by *Chlamydia trachomatis*
  □ **d.** Microbial keratitis should be treated with hourly broad-spectrum antibiotics
**Question 4 Which immediate management protocol is best for the eye emergencies listed to the right?**
  **a.** Chemical burn
  **b.** Orbital cellulitis
  **c.** Acute glaucoma
  **d.** Penetrating injury
  **e.** Posterior capsule rupture
  **f.** Microbial keratitis
  **i.** Intravenous antibiotics
  **ii.** Eye shield
  **iii.** Anterior vitrectomy
  **iv.** Intensive irrigation
  **v.** Hourly topical antibiotics
  **vi.** Acetazolamide 500 mg

## ANSWERS

1. a. False. It is everyone's responsibility. b. False. Practice means one is more prepared and better able to manage the real emergency. c. False. Some emergencies require immediate intervention before referral. d. True. Most health workers will encounter an eye injury or other ocular emergency at some time.
2. a. False. Retinal detachment does not cause a red eye, although loss of vision may be sudden when the macula becomes detached. b. True. In acute glaucoma the eye will be painful, red and hard. Visual loss is due to associated corneal oedema. c. True. A corneal infection will cause a painful red eye with loss of vision due to an ulcerated cornea. d. False. Orbital cellulitis can cause a red eye and loss of vision but would also cause swollen eyelids and, often, proptosis. e. False. In optic nerve compression the onset of visual loss is usually slow and the eye remains white, but the globe may be proptosed and the eyelids swollen and tense.
3. a. False. Once cultures have been taken, treatment for endophthalmitis with broad spectrum antibiotics should start. The sooner intravitreal antibiotics are given, the more likely the eye will recover. b. False. Orbital cellulits requires intravenous systemic antibiotics. c. False. It is caused by *Neisseria gonorrhoeae*. d. True
4. a. iv b. i c. vi. d. ii. e. iii. f. v

